# Supplementary material for: The Enhanced Brief Structured Observation Model: Efficiently Assess Trainee Competence and Provide Feedback
Source: MedEdPORTAL. 2021 May 5;17:11153. doi: 10.15766/mep_2374-8265.11153 (PMC8096882; doi:10.15766/mep_2374-8265.11153)
Supplement: Supplementary file 1 — Facilitators Preworkshop Orientation.docxFacilitators Guide.docxClinical Encounter Card.docxEvaluation Questionnaires.docx [file mep_2374-8265.11153-s001.zip › A. Facilitators Preworkshop Orientation.docx]

**Appendix A – Facilitator’s Pre-workshop Orientation of Medical Students and Residents to Their Roles**

Setting up and performing the observation represents the major focus of the workshop, i.e., establishing guidelines for students, residents, and faculty before entering the patient room, what happens in the room, and providing feedback post-observation. There will be a medical student portraying a patient presenting with an acute medical problem in a patient room (either a space in the assigned room for the workshop or an additional room for the breakout session when small groups of faculty, resident and SPs will be interacting).

**Preparation of the students**

- Email participating medical students the clinical case they will be portraying so they are familiar with the content
- Meet with the students for 10-15 minutes before the workshop to discuss the nuances of the portrayal of the simulated patient to address questions.
- Emphasize authenticity presenting as an acutely ill young adult; e.g., stay in role and answer questions that the resident might ask that are not scripted below as realistically as possible

**Resident role**

- Remind the resident that their purpose in the workshop will be to conduct an unrehearsed interview of the simulated patient presenting for an acute care visit.
- The resident will be doing the interview in the presence of a designated supervising attending physician, with other workshop faculty observing in the background.
- The resident’s interaction with the simulated patient will provide a practice opportunity for faculty to practice observing residents and providing feedback.
- The visit should replicate a “real life” encounter and the resident should obtain the history from the patient as they usually would.
- Notify the resident that they will benefit from the workshop in that they will receive reinforcing and constructive feedback after the observation.

**Role of Simulated Patient***:*

- Scenario: You are a young adult aged 18 years who presents with a three-day history of mild URI, no fever noted (temperature not taken), and now a sore throat for 2 days. Your glands in your neck feel swollen and tender to touch. You are more tired since the illness and unable to do schoolwork. You are a first-year college student and concerned your illness will affect your attendance and grades (DO NOT OFFER THIS UNLESS ASKED). You have taken over-the-counter Cepacol lozenges, saltwater gargles and Tylenol for your sore throat. You feel you are not improving and thus the reason for the visit. You have been previously well, with no chronic illnesses or significant problems in the past. You are sexually active (heterosexual, IF ASKED) but use protection and have not had any oral sex recently (DO NOT OFFER UNLESS ASKED). Your immunizations are up-to-date, including HPV vaccine. You have no allergies. You are on no medications.
- Specific Instructions: A resident will be taking your history. Reply only to the resident’s questions and DO NOT OFFER INFORMATION NOT REQUESTED. Stay in the role; i.e., talk with slightly muffled voice, act ill (no laughing or responding to comments not part of the reason for the visit), and act concerned. If you are asked a question that is not covered here, try to make up a reasonable answer.
