# Supplementary material for: The Enhanced Brief Structured Observation Model: Efficiently Assess Trainee Competence and Provide Feedback
Source: MedEdPORTAL. 2021 May 5;17:11153. doi: 10.15766/mep_2374-8265.11153 (PMC8096882; doi:10.15766/mep_2374-8265.11153)
Supplement: Supplementary file 1 — Facilitators Preworkshop Orientation.docxFacilitators Guide.docxClinical Encounter Card.docxEvaluation Questionnaires.docx [file mep_2374-8265.11153-s001.zip › B. Facilitators Guide.docx]

**Appendix B – Facilitator’s Guide: Script and Key Talking Points**

**Time Required: 60-75 minutes**

**Method:**

1. Workshop Introduction (20-25 minutes):
   1. Overview (10-15 minutes):
      1. In the brief introduction to the workshop, the facilitator provides an overview of the ACGME competencies as the “gold standard” for assessing and assuring the competency of medical trainees. Based on the competencies, there is a mandate for faculty to directly observe trainees’ performance for competency-based assessments in the context of milestones and entrustable professional activities.
         1. There are many reasons why faculty do not currently observe trainees on a regular basis, most importantly time requirements. Trainees continue to report that faculty do not observe them often.
         2. The workshop will provide faculty with a model that will enable them to observe trainees effectively and efficiently, while also teaching how to provide trainees feedback.
         3. The highlight of the workshop will be practice opportunities that will simulate real life situations.
      2. The facilitator provides some didactic background for the brief structured observation model as originally published by Pituch et al. and subsequently modified by us. The main teaching points of this model are that observations can be done in brief ‘snippets’ (2-10 minutes) followed by feedback on performance that averages five minutes or less.
         1. Pituch’s model, which was published as an abstract in 1999, focused on providing direct and timely feedback to trainees. In that model, faculty were directed to observe for 3-5 minutes (writing everything the trainee said in the interaction) and then provide feedback.
         2. We have revised and expanded this model, allowing for a range of observation time and precluding faculty from having to write all trainee comments in the patient interaction.
         3. In addition, we have added important segments to the model; i.e., how to set up an observation for faculty, conduct the observation, and debrief with the trainee post-observation to provide effective feedback utilizing the Clinical Encounter Card (CEC).
         4. In this workshop, we have also utilized simulation with actual trainees instead of faculty roleplay to provide a higher fidelity experience paralleling that in the clinical setting.
      3. It is important to convey to faculty that with time, trainees ‘expect’ observations to occur because they value them, and it is clear they do not interfere with the doctor-patient relationship.
   2. Objectives (5 minutes): The facilitator can post the workshop objectives on a chalkboard or PowerPoint slide and verbally reinforce them:
      1. To articulate the benefits of direct observation in assessing residents and how this can lead directly to improved feedback
      2. To help faculty understand why direct observation of trainees in the clinical setting is not done routinely
      3. To describe to faculty the background of brief structured observation (BSO), the principles of feedback, and practice using the clinical encounter card (CEC) to provide this feedback
      4. To apply principles of BSO and feedback in a practice situation utilizing simulation
      5. To reflect with faculty how this model can be used in their individual settings to better informing competency-based trainee assessments by increasing both the number of observations of trainees and the number of opportunities for feedback.
   3. Observation discussion (5 minutes):
      1. Discussing participants’ experiences with observations and barriers that they perceive. The purpose of this exercise is to ‘diagnose’ the learners and determine where they are regarding their experiences with observing. The prompting questions would include:
         1. What have been your experiences with observing residents?
         2. What barriers have you encountered in performing these observations?
      2. Responses one could expect based on conducting this part of the workshop include:
         1. With other responsibilities, faculty do not perceive there is enough time to observe trainees
         2. The ground rules for the interaction involving the resident, patient and attending physician are rarely, if ever, explicit and left to the faculty’s discretion; i.e., there is no model.
         3. There are concerns that the observation can interfere with the trainee-patient relationship, suggesting the patient might direct questions to the more senior person in the room.
         4. Some trainees are reticent to being observed and feel like they are constantly being evaluated. Using consistent observations over time helps to relieve these perceptions, especially when faculty explicitly state that the main goal of the observation was to make them better physicians.
         5. Faculty may be concerned that they are not expert in observing aspects of the doctor-patient interaction; e.g., communication skills..
         6. Observations may be intrusive if the nature of the visit is sensitive; e.g., sexually transmitted disease, mental health issue.
2. Clinical Encounter and Observation (10-15 minutes): Each group of participating faculty, numbering 4-8 people depending on the workshop attendance, will be observing the resident. One of that group will volunteer to be the supervising attending physician, with the others being active participants ready to provide suggestions and advice along the way during the observation and the feedback session. The attending physician will instruct the recruited resident to take an unrehearsed history from the simulated patient and that the attending will be observing the history. Prior to entering the room, the attending will request the resident to ask the patient permission for the observation, stating that the attending is their supervisor and there to observe the resident’s performance. The attending physician will instruct the resident to inform the patient only to address questions/comments to the resident.
3. Post-observation (20-30 minutes):
   1. Feedback process (10-15 minutes): After the interaction, the attending physician, along with other faculty in the group, will complete the CEC (Appendix C), discussing what each feels should be an essential part of the feedback to the resident. The supervising attending asks the resident to reconvene for feedback on the observed encounter. The attending will provide verbal feedback using the SOAP model and share the CEC written findings (reiteration of the verbal feedback) with the resident during that discussion.
   2. The feedback model is based on the SOAP model initially used for recording information in a systematic way for the medical record. The acronym is hence easy to remember and use:
      1. **S for Subjective**: ‘So, how did you think things went?’ If response is ‘I think I did well,’ the follow-up question could be ‘Tell me what you think you did well and what you think you need to work on?’.
      2. **O for Objective: ‘**This is what I observed while in the room with you. I noted that you did not wash your hands in front of the patient. You did a terrific job in including the child in the interaction.’
      3. **A for Assessment: ‘**This is what I see as the major teaching points around the visit.’ Provide a couple teaching points; e.g., ‘use more open-ended questions,’ or ‘make the counseling more interactive.’
      4. **P for Plan: ‘**Based on this interaction, what would you do next time to make a similar interaction even better?’ Include some concrete ways trainees reflect on how they can improve their performance. Faculty should be collaborative in helping the trainee around a specific objective they have identified. ‘We can work on this…just let me know when you see a patient with a questionable assessment of the tympanic membrane and I or my colleagues will validate your findings’.
   3. Post-feedback discussion (10-15 minutes): Following the feedback, the entire group will reflect on the BSO process in terms of its applicability, feasibility and usefulness. Questions that each group should reflect on include:
      1. What did everyone think of the usefulness of the brief structured observation? Start with the medical students and get their feedback. Then ask the residents followed by the faculty participating in the workshop.
      2. Is the feedback model appropriate for your institution, and if so, in what form would it be used?
      3. What did the group think about the strong and weak points of the observation?
      4. Do participants think this approach will enhance observations and feedback in the clerkship and residency programs?
      5. How will you get colleagues involved so as to catalyze the experience?
4. Questions and Evaluation completion (5 minutes)
