# Supplementary material for: The Enhanced Brief Structured Observation Model: Efficiently Assess Trainee Competence and Provide Feedback
Source: MedEdPORTAL. 2021 May 5;17:11153. doi: 10.15766/mep_2374-8265.11153 (PMC8096882; doi:10.15766/mep_2374-8265.11153)
Supplement: Supplementary file 1 — Facilitators Preworkshop Orientation.docxFacilitators Guide.docxClinical Encounter Card.docxEvaluation Questionnaires.docx [file mep_2374-8265.11153-s001.zip › D. Evaluation Questionnaires.docx]

**Appendix D -** **The Questionnaires Utilized to Evaluate the BSO Model**

[Adapted by authors; Brookfield S. Becoming a critically reflective teacher. San Francisco: Jossey-Bass; 1995]

**I. The Brookfield Critical Incident Questionnaire (CIQ)**

- At what moment did you feel most engaged in the workshop?
- At what moment did you feel most distanced in the workshop?
- What action that anyone took did you find most affirming or helpful?
- What action that anyone took did you find most puzzling or confusing?
- What about the teaching session surprised you the most?

**II.** **The questionnaire completed by pediatric residents in clinic**

- Reflect on your interaction with the observer and record your comments.
- What were the main teaching points you learned today?
