# Supplementary material for: The Enhanced Brief Structured Observation Model: Efficiently Assess Trainee Competence and Provide Feedback
Source: MedEdPORTAL. 2021 May 5;17:11153. doi: 10.15766/mep_2374-8265.11153 (PMC8096882; doi:10.15766/mep_2374-8265.11153)
Supplement: Supplementary file 1 — Facilitators Preworkshop Orientation.docxFacilitators Guide.docxClinical Encounter Card.docxEvaluation Questionnaires.docx [file mep_2374-8265.11153-s001.zip › Educational_Summary_Report_11153.pdf]

MedEdPORTAL® The AAMC Journal of Teaching and Learning Resources

Original Publication Open Access

The Enhanced Brief Structured Observation Model: Efficiently Assess Trainee Competence and Provide Feedback

Scott Baumgartner, MD*, Dewesh Agrawal, MD, Larrie Greenberg, MD

*Corresponding author: baumgartners2@upmc.edu

Abstract

Introduction: The regular observation of trainees is essential to ascertain trainee proficiency in competency-based assessments. Unfortunately, observation of residents is not frequent enough to facilitate entrustment decisions, and the busy clinician-educator may not have the tools or time to conduct effective and efficient observations. **Methods:** We created a hands-on faculty development workshop utilizing an enhanced variation of the brief structured observation (BSO) technique to train both primary care and subspecialty pediatric faculty on how to effectively and efficiently observe trainees. The workshop has provided faculty a practical approach to observing trainees in a focused fashion and providing effective feedback on clinical skills based on their observation. In the workshop, faculty had an opportunity to observe residents taking an unrehearsed history from a medical student simulating an acutely ill patient, culminating in feedback on the residents' performance using the subjective, objective, assessment, and plan (SOAP) format. **Results:** This faculty development workshop has been presented to more than 100 faculty both locally and nationally, and feedback has been uniformly positive, with three institutions incorporating this model into their programs to date. **Discussion:** This enhanced BSO workshop promotes a model that streamlines the observations of trainees and provides faculty with the tools to encourage more observations.

Keywords
Assessment, Competency-Based Medical Education, CBME, Faculty Development, Direct Observation, Structured Clinical Observation

Educational Objectives

By the end of this activity, faculty will be able to:

1. Articulate the importance and benefits of direct observation in assessing residents' performance.
2. Describe how direct observation, using the brief structured observation (BSO) model, improves quality of feedback.
3. Discuss the perceived barriers to direct observation and understand why faculty do not observe trainees more frequently.
4. Describe the background of BSO, the principles of feedback, and how to use the clinical encounter card to provide this feedback.
5. Apply principles of BSO and feedback in a practice situation utilizing simulation.
6. Reflect on how this model can be used in their individual settings to better inform competency-based trainee assessments by increasing both the number of observations of trainees and the number of opportunities for feedback.

Citation:
Baumgartner S, Agrawal D, Greenberg L. The enhanced brief structured observation model: efficiently assess trainee competence and provide feedback. *MedEdPORTAL*. 2021;17:11153.
https://doi.org/10.15766/mep_2374-8265.11153

Introduction

Performance-based measures designed to adequately assess competency and achievement of medical trainees require direct observation in the clinical environment.[1-4] Unfortunately, direct observation of trainees has not been demonstrated to be a priority in academic health centers, and there are few models demonstrating how it can best be accomplished. Balancing patient care and teaching responsibilities, clinical teaching faculty might not have, or not be able to make, time to directly observe trainees. In addition, many faculty have not been trained to efficiently and effectively observe trainees, nor do they always feel comfortable in assessing aspects of the trainee's performance and providing necessary reinforcing and constructive feedback. A literature search in PubMed (with the keywords *direct observation*, *structured clinical observation*, *students*, *medical students*, *undergraduate medical education*,

Copyright © 2021 Baumgartner et al. This is an open-access publication distributed under the terms of the Creative Commons Attribution license.

graduate medical education, *internship*, and *residency*) yielded 494 potentially relative articles that specifically assessed direct observation of medical trainees. Of these, only 26 articles discussed trainee competence and direct observation, and only nine of these 26 articles quantified direct observation in assessing competencies and milestones, while the remainder discussed these topics on a more conceptual and programmatic level. In searching for applicable models in *MedEdPORTAL*, two publications have addressed direct observation, one focusing on handoffs regarding patient care[5] and the other validating an instrument assessing professionalism, patient care, and communication.[6] However, neither of these publications provides details of the process of direct observation before, during, and after the observation occurs.

To facilitate and enhance the important process of faculty observation of trainees, we have developed a faculty development workshop using an enhanced variation of the brief structured observation (BSO) model.[7] The BSO model was originally created to provide "patient-friendly, learner-focused, and efficient feedback to residents and student in busy offices or wards" and involves a 2-minute observation by faculty, who recorded everything the trainee said in the interaction.[7] We have revised and expanded this model, allowing for a range of observation time and precluding faculty from having to write all trainee comments in the patient interaction. In addition, we have added important segments to the model, for example how to set up an observation for faculty, conduct the observation, and debrief with the trainee postobservation to provide effective feedback utilizing the clinical encounter card (CEC).[8] In this workshop, we have also utilized simulation with actual trainees instead of faculty role-play to provide a higher fidelity experience paralleling that of the clinical setting.

Methods

Development and Preparation

The enhanced BSO faculty-development workshop was directed at educational leadership and clinical faculty who were responsible for observing residents, fellows, or students. In the workshop, we provided not only a vision of the structural framework for faculty to balance trainee observation with clinical responsibilities, but we also enabled practice opportunities to observe residents conducting a clinical encounter while medical students portrayed a simulated patient. We have conducted the workshop for specific groups (e.g., divisions within a department or departments within the medical school) and advertised the training as a way to increase the number of trainee observations in an efficient and effective manner. In marketing the workshop to faculty, we acknowledged that time restraints play a major role in how often faculty are able to observe trainees during clinical encounters and how learning the techniques presented in the workshop will increase trainee observations, thereby enhancing assessment and providing a mechanism to provide more reliable feedback. Ultimately, we designed the enhanced BSO model and workshop to improve the feedback and competency assessment process for trainees.

Having busy academic clinical faculty attend this core faculty development workshop was an anticipated challenge. Planning tools (e.g., emails, flyers, etc.) were institution-specific in order to optimize and leverage institutional culture in attracting participants. We engaged division chiefs and departmental chairs to encourage their faculty to attend and used departmental monthly meeting time to conduct the workshop with better attendance. When possible, we offered workshops at different times and days to maximize participation. Importantly, we made clear that the workshop would provide hands-on experiences in order to challenge faculty to practice the skills outlined in the observation model.

In recruiting, we considered the optimal small group to be one simulated patient (a medical student portraying a young adult with an acute problem), one resident, and approximately six to eight faculty participants. It may be necessary to have multiple small groups to accommodate a larger group of faculty; however, each group should still seek to maintain this six to eight faculty per one resident per one simulated patient ratio to allow for rich peer feedback and input. If necessary, the number of faculty participants could be as few as four or five. However, smaller numbers may limit the quality of the peer discussion that the workshop fosters. We delegated recruitment efforts of residents and medical students to the residency training program and clerkship directors, informing them that the major goal of the workshop was to increase the number and quality of observations of trainees by providing a real-life experience for faculty, utilizing students and residents in a simulation. Having medical students portray a simulated patient has demonstrated improved performance in their high-stake OSCE exams and also was a learning experience for them.[9,10] Residents were often willing to participate once they understood that they would receive some benefit from this exercise by being provided feedback on their clinical skills.

Workshop Overview

The workshop included three segments: (1) preworkshop, (2) workshop didactics and observed clinical encounter, and (3) postobservation. The preworkshop activities (Appendix A)

Copyright © 2021 Baumgartner et al. This is an open-access publication distributed under the terms of the Creative Commons Attribution license.

provided an opportunity to ensure a successful workshop by promoting effective marketing communication and logistics, as well as resident and medical student (simulated patient) recruitment. The time required for this administrative effort was variable but decreased after each iteration.

The workshop didactics, content, and logistics of the observed clinical encounter are detailed in the facilitator guide (Appendix B). There was no preparation necessary for those faculty participating in the workshop. The main content areas of the workshop included a brief introduction followed by a discussion of objectives, barriers to observation, followed by the experiential and hands-on portion of the workshop. The latter involved a clinical BSO of a resident and a simulated patient, portrayed by a medical student, followed by the provision of feedback by the faculty to the resident on their demonstrated clinical competence. The workshop ended with a discussion of the applicability, feasibility, and usefulness of the BSO process and implementing it in the faculty's teaching roles. The allotted time for this workshop ranged from 60-75 minutes, with the slightly longer time enabling more discussion about the clinical encounter and competency feedback that should be provided.

Workshop Implementation

In the introduction to the workshop (20-25 minutes), we provided an overview of the utility of the ACGME competencies in assessing and assuring the competency of medical trainees, reviewed workshop objectives, discussed barriers to observation, and provided an overview of the workshop to participants. We explained that competency-based assessments mandate direct observations by faculty. We then explained how this workshop will provide busy faculty with a practical approach to observing trainees in a focused fashion and providing effective feedback on clinical skills based on that observation. We posted the workshop objectives on a chalkboard and verbally reinforced them. As an icebreaker, we asked faculty participants what their experiences have been observing residents and then transitioned this into a brief discussion of typically identified barriers. We then provided an overview of the workshop, discussed the principle of BSO, and formulated how we would use the CEC and deliver feedback to the resident in the simulated clinical encounter.

For the clinical encounter portion of the workshop (10-15 minutes), we solicited a volunteer from each of the faculty small groups to serve as the supervising attending during the clinical encounter. The role of the supervising attending was explained to the faculty participants, including that the supervising attending with the help of the faculty participants would be providing feedback to the resident after the clinical encounter. The other faculty in the small groups actively observed the clinical encounter in order to provide suggestions and advice to the supervising attending for the feedback session.

In the postobservation segment (20-30 minutes), the faculty small-groups briefly discussed which points they wanted to include in the feedback session to the resident. This reinforcing and constructive feedback was captured in writing by the faculty using the CEC (Appendix C). The CEC was chosen because it reflects a brief account of the feedback and has been used in other studies.[8] The participating faculty, led by the supervising attending volunteer, provided feedback to the resident, both verbally and using the CEC. The verbal feedback was based on the subjective, objective, assessment, and plan (SOAP) format (Appendix B), which offered a structure for the faculty's approach. The SOAP acronym,[11] based on a format to organize information in the medical record, is easy to remember, but other feedback models could be utilized. Both the CEC and SOAP instruments incorporate the adult learning principle of self-directed improvement.

This segment of the workshop continued with the opportunity to reflect on the BSO model itself with the participating students, residents, and faculty. Topics included the value of the BSO model and its applicability in different settings, perceptions of the CEC and its use for formative and summative feedback, and the ease and effectiveness of using the SOAP model for feedback. Finally, workshop participants completed the Brookfield Critical Incident Questionnaire[12] (CIQ; Appendix D) to assess the workshop's content globally and specifically with respect to applying the BSO model to their setting. Responses were recorded as free text. The Brookfield CIQ is a widely utilized tool for understanding what factors affect learning after a learning experience via narrative responses.[13]

Clinical Implementation

In addition to the workshop, the senior author (Larrie Greenberg) has utilized the enhanced BSO model to conduct real-life observations of pediatric residents in their continuity clinic at the Children's National Medical Center. To assess residents' experiences from these real-world interactions, we asked for anonymous written responses to two questions (Appendix D) on the residents' reflections and main learning points.

Assessment

We performed a semiqualitative analysis to identify themes from both the Brookfield CIQ completed during the workshops and the two questions posed to pediatric residents after BSO observations in continuity clinic.

Copyright © 2021 Baumgartner et al. This is an open-access publication distributed under the terms of the Creative Commons Attribution license.

Results

Workshop Feedback
The senior author (Larrie Greenberg) has conducted this workshop at our home pediatric institution, locally in the DC area at two medical schools, nationally at other medical schools as part of visiting professorships, and at national society meetings over the last 2 decades. Workshop participants have mainly, but not exclusively, been pediatric faculty (both primary care and subspecialty) and have totaled well over 100 people.

Feedback has been uniformly positive. Free-text feedback from workshop participants ($n = 44$) utilizing the Brookfield CIQ (five questions) is summarized by theme in Table 1. Faculty appreciated the opportunity to practice direct observation of resident clinical skills in the simulated environment and to practice providing structured feedback based on those observations with a helpful model.

Clinical Feedback
Feedback provided by pediatric residents ($n = 186$) at our institution following direct observation in the outpatient setting is also summarized by theme in Table 2. The overwhelming majority of residents found the observation model very helpful and were appreciative of the time devoted to the process of observation itself and to the feedback provided with key teaching points after every encounter.

Generalizability
To assess the impact and generalizability of this training, we are aware of at least three institutions where this workshop was provided that have subsequently incorporated our enhanced observation model into their educational programs in either the inpatient and outpatient settings, requiring trainees to obtain CECs after a BSO by a faculty member as part of their assessments.

Discussion

The enhanced BSO represents a model by which academic clinical faculty can observe trainees in an efficient and effective manner, provide timely and specific feedback, and record the summary on a CEC. Direct observation represents the major way to assess the professional competence of trainees.[1-6] With few models in the literature on which to base one's observations, the enhanced BSO model we have presented gave faculty a tool they can use to observe trainees more frequently and provided an opportunity to give more specific and effective feedback.

Our results demonstrated that this workshop has been well received, and that by utilizing the principles outlined, brief observations of trainees in real clinical scenarios can be facilely accomplished, and effective feedback can be provided through such direct observations. Importantly, the observation and feedback realistically can be performed in 10 minutes or less, in the midst of a routine clinical encounter. This economy of time in our model respected faculty's busy schedules and promoted observation as an important part of their responsibilities. The senior author (Larrie Greenberg) has used this model for decades and has demonstrated that by adhering to the principles we established in the model, faculty can perform a greater number and more effective observations in a limited time period. These

Table 1. Summary of Faculty Evaluations of the Brief Structured Observation Model in the Workshop ($n = 44$)

Themes	Quotes
Observation model	"Participating in the session when the resident interacted with the simulated patient."
	"I was happy to see the feedback part of the model in written form (CEC)."
	"...The fact that you could observe and give feedback in a very short period of time (6.5 minutes)."
	"Brevity and ease of the tool."
	"Setting up the expectations for the observation, the SOAP model, brevity of the model, use of the CEC for assessment."
Feedback process	"Receiving the feedback in such a well organized way."
	"How easy it was to give the feedback."
	"I was more confident in giving feedback than I thought I would."
	"Feedback from the students about what they really want in terms of feedback from attendings."
	"I was most engaged using the tool to provide real-time feedback."
	"How to provide constructive feedback."
	"How simple and broadly applicable the card (CEC) can be."
	"How common sense the SOAP acronym is and how easy it is to implement."
Take-home learning points	"The residents' self-assessment was helpful; his level of insight was enlightening."
	"The model made me think more as faculty about my own interactions with patients."
	"I will take this model back to my division."
	"Figuring out how well an objective observation can go."
	"Didn't realize that such a model can be so short."
	"Could have been linked to the competencies and milestones."

Abbreviations: CEC, clinical encounter card; SOAP, subjective, objective, assessment, and plan.

Copyright © 2021 Baumgartner et al. This is an open-access publication distributed under the terms of the Creative Commons Attribution license.

Table 2. Summary of Resident Evaluations of the Brief Structured Observation Model in the Clinic (n = 186)

Themes	Quotes
Observation model	"I liked having a senior attending observing my counseling ...it gave me additional confidence."<br>"I was worried that the observation would be disruptive but having an observer was natural. It felt like a collaborative learning environment."<br>"He took time to know my name and ask me questions, which goes a long way for me to trust his advice."<br>"He made the process comfortable and gave good actionable advice."<br>"It's always nerve-wracking to have someone observe you; however, I always learn a lot. It's hard to see some of your strengths and weaknesses and this allows an objective way to do it."
Feedback process	"Great job discussing motivational interviewing and what we would discuss before interacting with the patient. It made it a live learning experience."<br>"Gave me detailed feedback on the encounter and covered all points."<br>"Able to help me during the interview without seeming too bossy or authoritarian."<br>"He took time to sit with me afterward. His feedback was supportive, gentle and caring with constructive comments."<br>"Gave strong advice on how to improve."
Take-home learning points	"He showed me how to do the cover/uncover test for strabismus."<br>"Introduced the concept of teach-back as to how family members would explain diagnosis and treatment to members not there."<br>"Be certain to undress the child in a sick visit."<br>"Allowed for safe space for parents to share their concerns and to ask if there was anything mentioned the parents were unable to do."<br>"Eliciting parents' main concern and address it."<br>"Taught me the testicular exam regarding a full versus an asymmetric scrotum."

observations are essential to informing competency-based performance assessments.

Lessons Learned

Lessons learned from using this model include the importance of setting up the ground rules for successful observation, establishing trust with trainees, and assuring them that the intent of the observations and provided feedback is to help them become better physicians. This trust does not happen automatically and requires the support of educational and clinical leadership to emphasize the importance of these observations. When implementing the BSO model it is necessary for trainees to be informed ahead of time. Ideally, during the clinical rotation orientation, faculty would make trainees aware that there will be opportunities for them to be observed over the course of the rotation, and that the intent of these observations is to target feedback on improving their performance. Trainees should understand that direct observation by supervisors helps to identify what trainees do well and what they need to improve.

Limitations

There are some limitations we have observed with the model. An important limitation is that we have based our model on the ambulatory general pediatric setting and have not applied it to other settings. It is likely that this model would be easily transferrable to any ambulatory setting, but we have not formally evaluated it in the operating room, preoperative area, or the inpatient setting. Another limitation is the relatively small number of completed CIQs received from the completed workshops. Also, there are few long-term outcomes reported from effective faculty development programs. Short-term gains are easy when participants value the exercise. Assessing longer-term impact is dependent on evaluation completion, which was not possible at the institutions where the senior author was invited to present this workshop. However, the subsequent utilization of our enhanced observation technique at other institutions pointed to the generalizability of our approach. This model is currently used in the pediatric clerkship at another large pediatric center with over 70 clinical faculty, where the 100 medical students who rotate each year are each required to obtain five CECs.

Another possible limitation was that we utilized the CEC and SOAP to model the feedback provided; however, other familiar feedback models would likely be substitutable. Although the CEC and SOAP approach to feedback do not directly address ACGME competencies or milestones, repeated use of the BSO approach with medical trainees will afford faculty the direct observations necessary to better inform these performance-based assessments. A final limitation was that our method of workshop analysis using the Brookfield CIQ did not allow us to quantitively assess whether we achieved every learning objective from our workshop, especially those at the lowest level in Bloom's taxonomy (i.e., knowledge and comprehension).

Future Directions

The expanded use of our observation model has the potential to assure the accurate assessment of trainee competence as they progressively acquire the essential milestones associated with their specific training. Whereas the original BSO model was created prior to the advent of ACGME competencies

Copyright © 2021 Baumgartner et al. This is an open-access publication distributed under the terms of the Creative Commons Attribution license.

and milestones, the utility of these observations of trainees performed by faculty cannot be underestimated within the current competency-based assessment paradigm. Further, the ability of this enhanced BSO model to inform important trainee entrustment decisions should be formally assessed.

Appendices

A. Facilitators Preworkshop Orientation.docx

B. Facilitators Guide.docx

C. Clinical Encounter Card.docx

D. Evaluation Questionnaires.docx

All appendices are peer reviewed as integral parts of the Original Publication.

Scott Baumgartner, MD: First-Year Resident, Department of Internal Medicine, University of Pittsburgh Medical Center; ORCID: https://orcid.org/0000-0002-4745-4021

Dewesh Agrawal, MD: Professor of Pediatrics and Emergency Medicine, The George Washington University School of Medicine and Health Sciences; Vice-Chair for Medical Education, Children's National Medical Center

Larrie Greenberg, MD: Professor Emeritus of Pediatrics, The George Washington University School of Medicine and Health Sciences and Children's National Medical Center

Acknowledgments
The authors would like to thank Maria Blanco, PhD, for her suggestions on revisions to this manuscript.

Disclosures
None to report.

Funding/Support
None to report.

Ethical Approval
Reported as not applicable.

References

1. Donato AA. Direct observation of residents: a model for an assessment system. *Am J Med*. 2014;127(5):455-460. https://doi.org/10.1016/j.amjmed.2014.01.016
2. Kogan JR, Holmboe ES, Hauer KE. Tools for direct observation and assessment of clinical skills of medical trainees: a systematic review. *JAMA*. 2009;302(12):1316-1326. https://doi.org/10.1001/jama.2009.1365
3. Goch AM, Karia R, Taormina D, et al. A comparison of assessment tools: is direct observation an improvement over objective structured clinical examinations for communications skills evaluation? *J Grad Med Educ*. 2018;10(2):219-222. https://doi.org/10.4300/jgme-d-17-00587.1
4. Smith J, Jacobs E, Li Z, Vogelman B, Zhao Y, Feldstein D. Successful implementation of a direct observation program in an ambulatory block rotation. *J Grad Med Educ*. 2017;9(1):113-117. https://doi.org/10.4300/jgme-d-16-00167.1
5. Starmer AJ, Landrigan C, Srivastava R, et al. I-PASS Handoff Curriculum: faculty observation tools. *MedEdPORTAL*. 2013;9:9570. https://doi.org/10.15766/mep_2374-8265.9570
6. Swan R, Gigante J. Direct observation in an outpatient clinic: a new easier tool. *MedEdPORTAL*. 2010;6:7901. https://doi.org/10.15766/mep_2374-8265.7901
7. Pituch K, Harris M, Bogdewic S. The brief structured observation—a tool for focused feedback. *Acad Med*. 1999;74(5): 599. https://doi.org/10.1097/00001888-199905000-00073
8. Ozuah PO, Reznik M, Greenberg L. Improving medical student feedback with a clinical encounter card. *Ambul Pediatr*. 2007;7(6):449-452. https://doi.org/10.1016/j.ambp.2007.07.008
9. Mavis B, Turner J, Lovell K, Wagner D. Faculty, students, and actors as standardized patients: expanding opportunities for performance assessment. *Teach Learn Med*. 2006;18(2): 130-136. https://doi.org/10.1207/s15328015tlm1802_7
10. Sasson VA, Blatt B, Kallenberg G, Delaney M, White FS. "Teach 1, do 1 ... better": superior communication skills in senior medical students serving as standardized patient-examiners for their junior peers. *Acad Med*. 1999;74(8):932-937. https://doi.org/10.1097/00001888-199908000-00020
11. Blatt B. Giving honest feedback: resident-to-student communication. *Virtual Mentor*. 2005;7(8):564-568.
12. Brookfield SD. *Becoming a Critically Reflective Teacher*. Jossey-Bass; 1995.
13. Keefer JM. The Critical Incident Questionnaire (CIQ): from research to practice and back again. Paper presented at: Adult Education Research Conference; May 28-30, 2009; Chicago, IL. http://newprairiepress.org/aerc/2009/papers/31

Received: January 11, 2021
Accepted: March 8, 2021
Published: May 5, 2021

Copyright © 2021 Baumgartner et al. This is an open-access publication distributed under the terms of the Creative Commons Attribution license.
