# Supplementary figures and images for: The Enhanced Brief Structured Observation Model: Efficiently Assess Trainee Competence and Provide Feedback
Source: MedEdPORTAL. 2021 May 5;17:11153. doi: 10.15766/mep_2374-8265.11153 (PMC8096882; doi:10.15766/mep_2374-8265.11153)

**Appendix C - Clinical Encounter Card (author owned)**
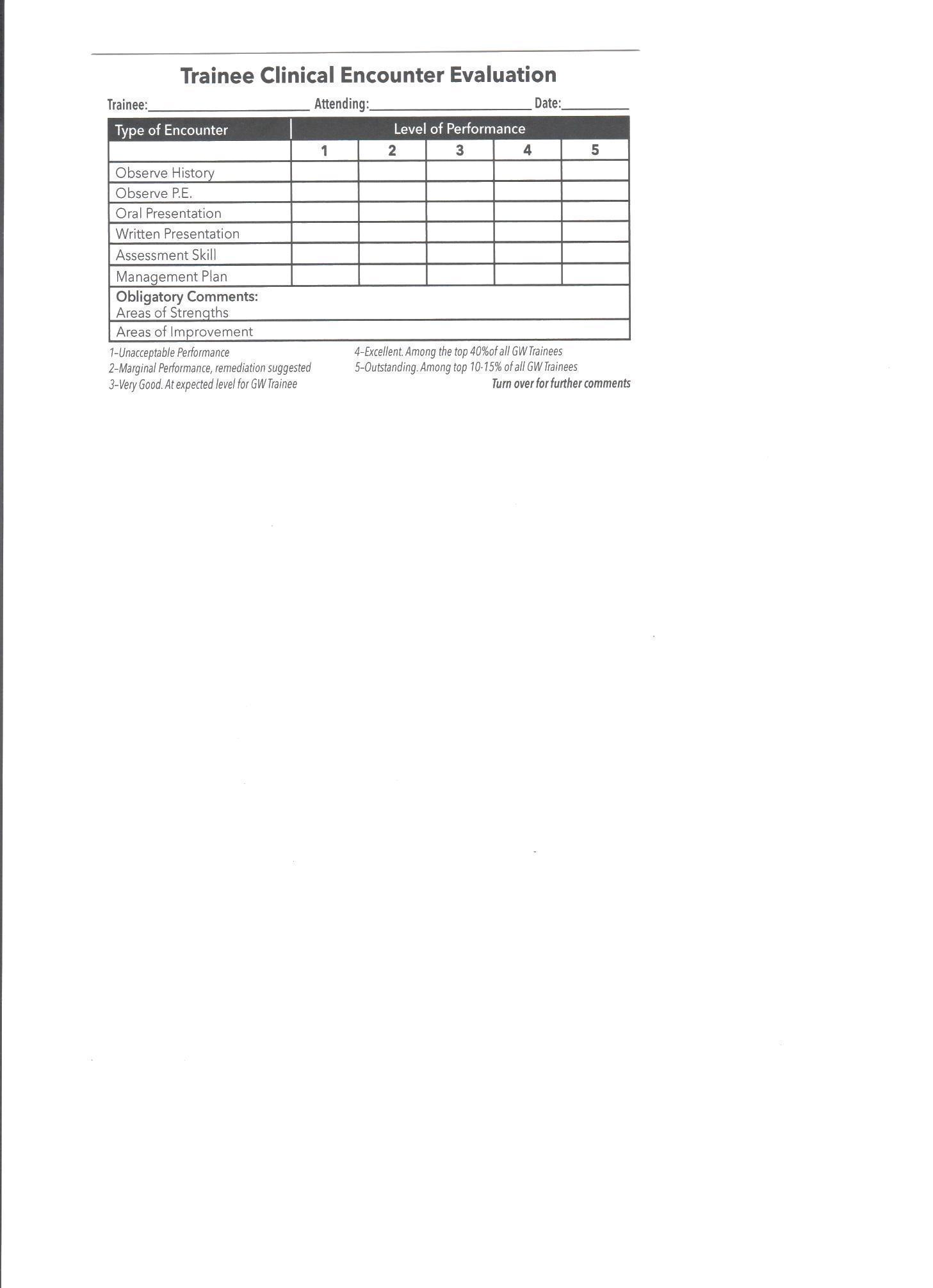


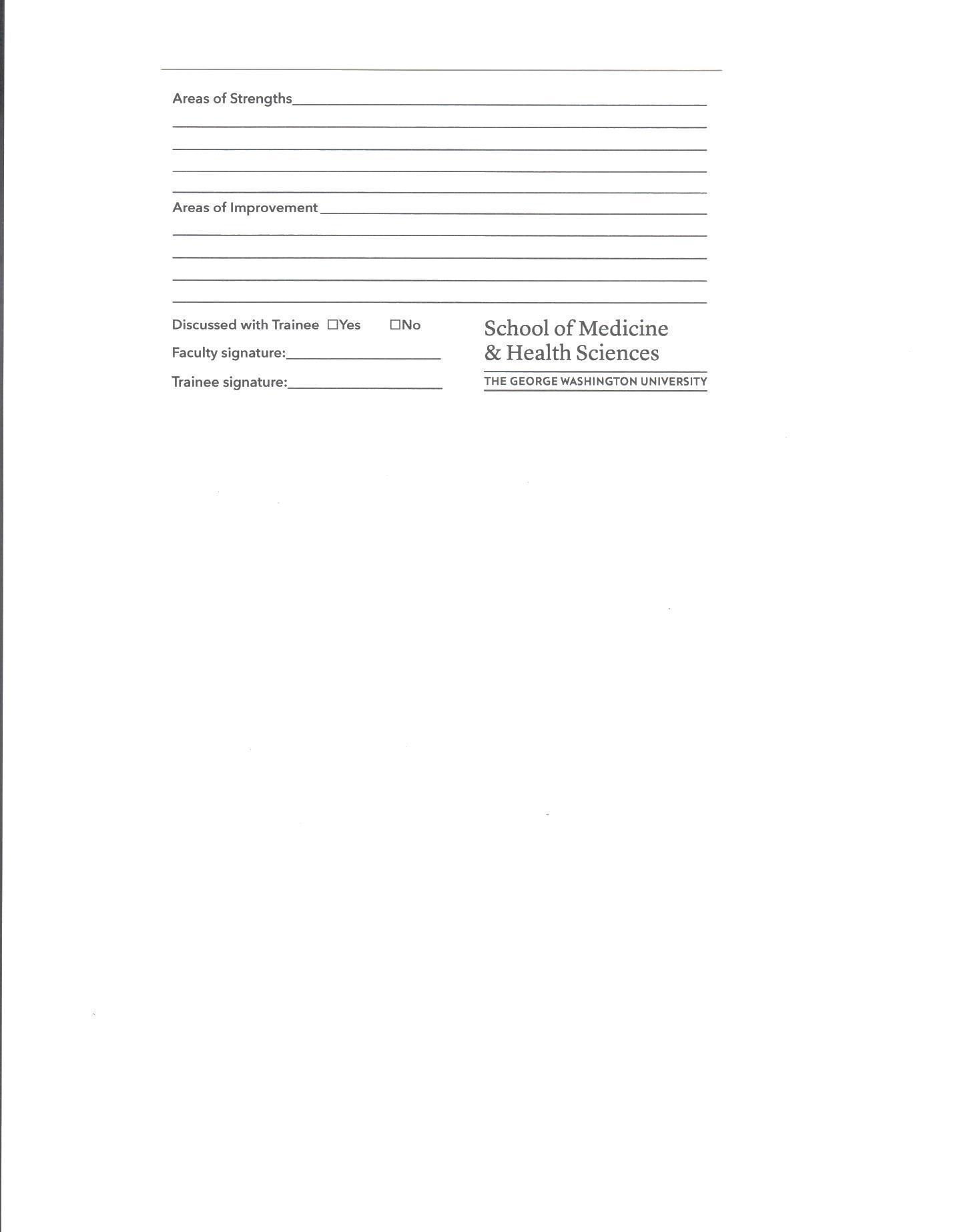

Supplement: Supplementary file 1 — Facilitators Preworkshop Orientation.docxFacilitators Guide.docxClinical Encounter Card.docxEvaluation Questionnaires.docx [file mep_2374-8265.11153-s001.zip › C. Clinical Encounter Card.docx]
